# Enhancing drug therapy in ostomy patients: Best practice recommendations for medication management

**DOI:** 10.1371/journal.pone.0305047

**Published:** 2024-06-06

**Authors:** Vivien Berger, Matthias Reeh, Martin Scherer, Steffen Härterich, Sven Möller, Eva-Maria Anette Wansing, Annika van der Linde, Claudia Langebrake

**Affiliations:** 1 Hospital Pharmacy, University Medical Center Hamburg-Eppendorf, Hamburg, Germany; 2 Department of General, Abdominal, Thoracic and Vascular Surgery, Katholisches Marienkrankenhaus, Hamburg, Germany; 3 Department of General Practice and Primary Care, University Medical Center Hamburg-Eppendorf, Hamburg, Germany; 4 Department of Urology, University Medical Centre Hamburg-Eppendorf, Hamburg, Germany; 5 Department of Stem Cell Transplantation, University Medical Center Hamburg-Eppendorf, Hamburg, Germany; Acibadem Maslak Hospital: Acibadem Maslak Hastanesi, TURKEY

## Abstract

**Background:**

Ostomy surgery is a common procedure that poses various challenges for patients and healthcare professionals. There are numerous guidelines addressing different ostomy-related problems (ORPs) and supporting an interdisciplinary approach for ostomy care, but evidence-based literature for optimizing drug therapy after ostomy surgery is lacking.

**Aim:**

To investigate and characterize typical ORPs in relation to drug therapy and provide best practice recommendations from a pharmaceutical point of view.

**Methods:**

Patients with an ileo- or colostomy were consecutively enrolled in a prospective, interventional monocentric cohort study during hospitalization, with particular attention to medication. A clinical pharmacist assessed DRPs by performing level 3 medication reviews and patient interviews. Pharmacists’ interventions (PIs) were evaluated by two senior clinical pharmacists and documented in DokuPIK (Documentation of Pharmacists’ Interventions in the Hospital). Following interdisciplinary discussions, physicians either accepted or rejected the proposed changes in drug therapy. Comparisons were made between ileostomy and colostomy patients regarding type and extent of PIs.

**Results:**

Out of the 80 patients included in the cohort, 54 (67.5%) had an ileostomy and 26 (32.5%) a colostomy. In this study, 288 PIs were documented (234 ileostomy *vs*. 54 colostomy), of wich 94.0% were accepted and implemented by the physicians. The most common reason for PIs in both subgroups (29.6% ileostomy *vs*. 26.1% colostomy) was a missing drug although indicated (e.g. no loperamide, but high stoma output). The proportion of PIs associated with the ostomy was higher in ileostomy patients (48.3% ileostomy *vs*. 31.5% colostomy; p = 0.025). Typical ORPs were extracted and analyzed as case studies including recommendations for their respective management and prevention.

**Conclusion:**

This study highlights the importance of clinical pharmacists being a part of interdisciplinary teams to collaboratively improve ostomy care and patient safety. Especially ileostomy patients are more vulnerable for ORPs in the context of drug therapy and need to be monitored carefully.

## Introduction

Have you ever been confronted with the problem that a tablet appeared undissolved in the ostomy bag of an ostomy patient? In this case, what have you done? Do you remember which drug it might have been? Did you find a solution for this problem?

In some cases, these questions reach clinical pharmacists–whether it is a phone call of the surgeon/physician, the WOC (Wound, Ostomy and Continence) nurse, nurses in general, dietitians or the patient himself who is facing this problem. If a whole tablet becomes visible in the ostomy bag, liberation and absorption of the drug is very likely to be impaired. As a result, the drug therapy can lose its effect. Especially during the first weeks and months after ostomy surgery, complications occur more frequently [1, 2].

The limited absorption of drugs in ostomy patients is a relevant problem which is often underestimated as it is rarely detected plus there is a lack of awareness. In contrast, there are international guidelines providing recommendations on the supplementation of nutrients and vitamins [3–6] and nutrition counseling representing an essential part of ostomy care [7, 8].

According to Nightingale et al., especially in patients with a short bowel syndrome, malabsorption of vitamins and nutrients as well as a reduced absorption of drugs needs to be considered [9]. Zanni et al. concluded that jejunostomy and ileostomy patients are more likely to have drug-related problems regarding to drug absorption than colostomy patients [10]. There are some studies and reports that have attempted to point out the absorption problems in ostomy patients [9–16], but evidence-based literature for drug therapy after ostomy surgery is still lacking. The Association of Stoma Care Nurses has developed a summary of guidelines addressing common complications and challenges regarding ostomy care [17]. The summary refers in different sections to the need for daily medication reviews.

Pharmacists encounter ostomy patients in both inpatient and outpatient settings, but the information about the patient’s ostomy and potential absorption problems are often insufficient. By improving the flow of information and involving pharmacists more actively, they can provide added value in terms of drug therapy. This has been recognized by the Registered Nurses’ Association of Ontario (RNAO), which names pharmacists as key members of the interprofessional team in their best practice guideline for ostomy care [18]. For comprehensive ostomy care, patients need access to expert healthcare professionals. While other healthcare professionals are already involved in ostomy care, there is still a lack of cooperation with clinical pharmacists. In recent years, various professional societies have recognized the importance and impact of involving clinical pharmacists in interdisciplinary teams. This is particularly true for areas such as emergency departments, intensive care units, stem cell transplantation and as a member of the antimicrobial stewardship program [19–22]. Clinical pharmacists can identify and solve drug-related problems (DRPs) through pharmacists’ interventions (PIs), thereby improving patient safety [23–29].

The aim of the study was to systematically investigate DRPs in ostomy patients and evaluate the potential impact of PIs within the subgroups of ileo- and colostomy patients. The primary objective was to raise awareness among healthcare professionals regarding absorption problems, to take action, adapt drug therapy and improve patient safety. In order to enhance drug therapy in ostomy patients, we aimed to provide useful recommendations for the management and prevention of typical DRPs.

## Material and methods

### Setting and study design

This prospective, interventional cohort study was conducted at the University Medical Center Hamburg-Eppendorf (UKE), Germany. The study was approved by the local ethics committee of the Ärztekammer Hamburg (2021-100645-BO-ff) and registered within the German Clinical Trials Register (DRKS 00027291).

We consecutively included adult ileo- or colostomy patients with an inpatient stay between February 14, 2022 and March 16, 2023. The study participants either had an existing ostomy or a new ostomy was created during the hospital stay. Further inclusion criteria were age ≥ 18 years, written informed consent as well as sufficient German language skills. The aim of the study was to improve the drug therapy for ostomy patients through an intensified pharmaceutical medication management. As the primary outcome we defined the extent of PIs to solve DRPs. Secondary endpoints were the classification of DRPs as well as the assessment of pharmaceutical management from the patients’ perspective.

The medication process at the UKE is referred to as Closed Loop Medication Management (CLMM) consisting of four elements [30]. In the first step, physicians prescribe medications in the electronic prescribing software (computerized physician order entry with clinical decision support: CPOE/CDSS). These prescriptions are reviewed and validated by clinical pharmacists. Afterwards, the medications are packaged individually for each patient in the hospital pharmacy as part of the unit-dose logistics. As a final step, the administration of the medications is documented in the electronic prescribing software. Within the CLMM, clinical pharmacists perform a large number of medication reviews every day. In this study, in addition to the routine process, a clinical pharmacist performed level 3 medication reviews [31] for ostomy patients during their hospital stay, with a specific focus on ostomy-related problems (ORPs). Proposals for therapy modifications were discussed interdisciplinary with physicians and the WOC nurse. As part of an individual comprehensive consultation, patients were informed about modifications of their drug therapy and special aspects of drug formulations in the context of ostomy therapy in general. To evaluate the outpatient setting patients received a phone call at two points–one week and three months after discharge. From the patient’s point of view, pharmaceutical management was surveyed by a pseudonymized questionnaire (online or handwritten). The results of the questionnaire and the challenges in the outpatient setting will be presented in a separate publication. This paper focuses on the results of an intensified medication management during inpatient care and resulting recommendations for the prevention and management of typical ORPs in clinical practice.

### Patient and medication data

For the characterization of the patient population, demographic and clinical data including age, sex, type of ostomy (ileo- *vs*. colostomy and temporary *vs*. permanent), diagnoses, surgical procedures, date of surgery and length of hospital stay were assessed. All patient data were collected from the electronic patient record system Soarian Clinicals® (Cerner Health Services Deutschland GmbH, Berlin, Germany, version 4.5.200). Additional information, including serum electrolytes (creatinine, potassium, sodium and magnesium), was accessible in the patient’s record for the medication review.

Both home medication and inpatient prescriptions were evaluated by drug, ATC (Anatomical Therapeutic Chemical) classification, dosage, interval, route of administration, formulation and duration. The medication data were retrieved from the electronic prescribing software ID MEDICS® (ID Information und Dokumentation im Gesundheitswesen GmbH & Co. KgaA, Berlin, Germany, version 7.8.39).

The home medication was obtained from at least two different sources of information: Scanned medication lists, previous hospital discharge reports, information from the community healthcare provider, and always verified by a patient interview.

### Pharmacists’ interventions (PIs)

For all PIs, the involved drug(s), the reason for PIs (e.g. dose adjustment, initiation/discontinuation, modification of the dosage form), the resulting action and the acceptance rate were documented according to the DokuPIK criteria (Documentation of Pharmacists’ interventions [32]).

Furthermore, we classified PIs into two categories: (a) ostomy-related or (b) regular (independent of an existing ostomy). The classification into ostomy-related PIs was based on a list of drugs from a previous project (see S1 Table). The list includes commonly used drugs for ORPs [33], along with specific reasons for PIs: advice on the selection and dosage or evaluation of a (no longer) existing indication for these drugs. Irrespective of the drug, PIs were assessed as ostomy-related if changing a drug formulation for better absorption was recommended. The classification into (b) regular refers to all PIs that were independent of the ostomy (e.g. discontinued home medication or necessary dose adjustments). The classification of PIs was reviewed by two independent senior clinical pharmacists and consensus was reached.

### Definitions

- **Anatomical Therapeutic Chemical (ATC) Classification**

The ATC classification is a hierarchical system by the World Health Organization (WHO) to divide the active substances of drugs into different groups according to the organ or system which they affect. There are five different levels from the main anatomical group (1^st^ level) to the chemical substance (5^th^ level) [34].

- **Drug-related problems (DRPs)**

Drug-related problems are events or circumstances relating to drug therapy that actually or potentially interfere with the intended outcome and can cause harm to the patient [35].

- **High Output Syndrome (HOS)**

A small bowel ostomy output greater than 1.5 L to 2.0 L within 24 hours is usually considered as a high output syndrome (HOS) [9]. The consequences of a HOS are electrolyte and fluid imbalance up to acute renal failure in severe cases [36]. The type of ostomy, the amount and composition of enteral intake and the volume of gastrointestinal secretion are relevant factors influencing the ostomy output.

- **Medication review**

A medication review is a structured analysis of the patient’s drug therapy. The aim is to identify and manage drug-related problems (DRPs) to increase effectiveness and minimize potential risks associated with drug therapy [37]. In the present study, medication review is classified as level 3 (clinical medication review). In addition to medication and clinical data, there is a face-to-face collaboration between the clinical pharmacist, physicians, nursing staff and the patient [31].

- **Ostomy-related problems (ORPs)**

Ostomy-related problems generally refer to all problems that may occur in association with ostomy care, from physical (e.g. peristomal skin complications, constipation, sexual problems) to psychological changes in everyday life [38]. In this study, ostomy-related problems mainly refer to drug therapy from a pharmaceutical point of view, considering the absorption site and drug formulations.

- **Pharmacists interventions (PIs)**

Regarding DokuPIK PIs are defined as "any communication/action solving and/or avoiding DRPs" and includes the "management of existing DRPs as well as any proactive approach avoiding potential DRPs within the medication use process" [39]. (a) Physicians can accept PIs as a proposal and consequently implement them, (b) they can reject PIs for reasons of risk-benefit assessment or non-acceptance or (c) the outcome is not known.

### Statistical analysis

Data were analyzed anonymously using MS Excel (Microsoft Corporation, Redmond, United States, version 2016) and IBM® SPSS Statistics (IBM, Armonk, United States, version 27). Patient characteristics were summarized using median and range. Percentages and frequencies were calculated to characterize prescribed drugs and the associated ATC classification system (considering the 1^st^ and 3^rd^ levels). The acceptance rate of PIs was calculated based on all PIs except those, where only information was provided to physicians, nurses or patients. For group comparisons, categorical variables were examined using chi-square text (χ^2^) or Fisher’s exact test. Continuous variables were expressed as the median and range and compared using the Mann-Whitney U test. The level of significance was defined as α = 0.05.

## Results

### Patient characteristics

A total of 80 patients were recruited and included in the study, comprising 54 ileo- and 26 colostomy patients. The median age was 63 years with a range of 26 to 84 years. Females accounted for 54.0% of patients. There were no significant differences regarding age and sex between the ileostomy and the colostomy subgroup.

In almost two-thirds of patients, there was a surgical indication for new ostomy formation within the current hospital stay. In 74.0% of these patients, surgery was performed because of a cancer diagnosis. While the reasons for surgical indications were similar, the incidence of new ostomies was found to be significantly higher in the ileostomy group than in the colostomy group (*p* = 0.04). The median number of home medication was three (range 0 to 19) and raised to nine prescriptions (range 3 to 19) during the hospital stay.

Further comparability data is shown in Table 1.

**Table 1:** Patient characteristics differentiated according to the type of ostomy.

	Ileostomy (*n* = 54)	Colostomy (*n* = 26)	*p* value
Age, years [median (range)]	62.5 (26–84)	63.1 (36–83)	0.26
Sex [male; female]	30; 24	13; 13	0.64
Ostomy [new formation; existing]	38; 16	12; 14	**0.04**
Surgical indications for new ostomy formation [n = 50]			
Cancer	27	10	0.48
Inflammatory bowel disease	5	1	1.00
Other	6	1	1.00
Length of hospital stay, days [median (range)]	15 (2–117)	12.5 (2–116)	0.59
Number of drugs			
inpatient prescriptions1, n [median (range)]	8.5 (4–19)	9.5 (3–19)	0.50
home medication at admission, n [median (range)]	2.5 (0–19)	4.5 (0–14)	0.23

^1^Including continued/ongoing home medication and on-demand medication, if administered within the last 24 hours.

### Medication data and pharmacists’ interventions

The prescribed drugs were cumulated for the entire hospital stay and classified according to ATC groups. Table 2 summarizes the most relevant ATC groups 1^st^ level and the associated 3^rd^ level subgroups with the most frequently prescribed drugs. In relation to the ATC group, the proportion of patients of the total study population treated with at least one drug was assessed. Within each ATC group, the two most common drugs were evaluated. For example, within the ATC subgroup "N02B Other analgesics" and antipyretics, 72 patients (90.0% of the total study population) were treated with at least one drug of this subgroup. Here, the top two drugs were metamizole sodium for 68 and paracetamol for 40 patients.

**Table 2:** TOP 4 ATC groups 1st level and the associated 3rd level subgroups with the two most frequently prescribed drugs.

Drug groups (ATC Classification System)	*n* patients (%)	Most frequent drugs (*n* patients)
**A**–**Alimentary tract and metabolism** *A02B* Drugs for peptic ulcer and GORD^1^ *A04A* Antiemetics and antinauseants *A06A* Drugs for constipation	71 (88.8) 45 (56.3) 42 (52.5)	Pantoprazole (66), Esomeprazole (22) Ondansetron (38), Dimenhydrinate (23) Macrogol, combinations (28), Sodium picosulfate (13)
**N**–**Nervous system** *N02B* Other analgesics and antipyretics *N02A* Opioids *N05C* Hypnotics and sedatives	72 (90.0) 65 (81.3) 38 (47.5)	Metamizole sodium (68), Paracetamol (40) Piritramide (42), Oxycodone (40) Zopiclone (27), Melatonin (10)
**B**–**Blood and blood-forming organs** *B05B* i.v. solutions^2^ *B01A* Antithrombotic agents *B05X* i.v. solution additives^2^	60 (75.0) 76 (95.0) 38 (47.5)	Electrolytes (59), Combinations (24) Enoxaparin (75), Acetylsalicylic acid (12) Combination of electrolytes and trace elements (26), Potassium chloride (24)
**J–Antiinfectives for systemic use** *J01D* Other beta-lactam antibacterials *J01X* Other antibacterials *J01C* Beta-lactam antibacterials, penicillins	52 (65.0) 43 (53.8) 26 (32.5)	Ceftriaxone (26), Meropenem (22) Metronidazole (44), Vancomycin (14) Piperacillin and BLI^3^ (15), Ampicillin and BLI^3^ (11)

^1^ Gastro-Oesophageal Reflux Disease

^2^ B05B: i.v. solutions include solutions for parenteral nutrition or for the electrolyte balance.

B05X: Additives are concentrated solutions containing electrolytes, vitamins or amino acids for correcting electrolyte balance

and nutritional status.

^3^ BLI = Beta-lactamase inhibitor

## Classification of drugs

### Classification and acceptance of PIs

A total of 288 PIs, including 234 in the ileostomy and 54 in the colostomy group were carried out as part of the pharmaceutical medication management focusing on ostomy. Overall, the intervention rate was 3.6 PIs per patient. Considering the different ostomy types, the intervention rate in the ileostomy group was 4.3 PIs per patient versus 2.1 PIs in the colostomy group (*p* = 0.006).

The five most frequent reasons for PIs are listed in Table 3 and refer to the main categories "drugs", "other" and "dose". The most common reason accounting for a quarter of all PIs (26.4%) was performed, because a drug was not prescribed even though there was an indication. Altogether, the top five reasons were responsible for more than two-thirds of the PIs (68.8%). All other reasons were involved in less than 5.0% of the PIs. The overall acceptance rate for all PIs was 94.0%, with the highest acceptance rate in the category "other" at 100.0% and the lowest in the category "drugs" at 91.4%.

**Table 3:** Top 5 reasons for PIs sorted by main categories according to DokuPIK.

Reasons for PIs by main categories and subcategories	*n* (%)	Acceptance^a^ [%]
Category **Drug** (Clear) indication, but no drug prescribed Inappropriate (or not most suitable) drug formulation in terms of indication (Clear) indication not (or no longer) given	**175 (60.8)** 76 (26.4) 43 (14.9) 31 (10.8)	**91.4** 97.3 90.7 80.6
Category **Other** Patient counselling or education	**58 (20.1)** 30 (10.4)	**100.0** 100.0
Category **Dose** (Inappropriate) dose	**49 (17.0)** 18 (6.3)	**97.9** 94.4
Top five subcategories	198 (68.8)	92.8
**Total**	**288 (100.0)**	**94.0**

^a^Acceptance rate of PIs refers to *n* = 266, excluding PIs where only information was provided to physicians, nurses or patients.

### Ostomy-related PIs

**Fig 1:**
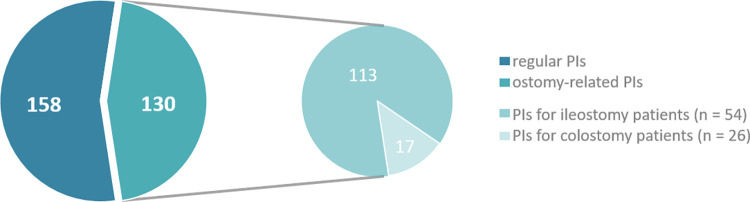
Classification of PIs among the ostomy subgroups ileo- and colostomy.

Fig 1 illustrates the distribution of PIs (n = 288) among the subgroups of ileo- and colostomy patients. More than half of the PIs (n = 158; 54.9%) were classified as regular PIs (ostomy-independent). Within the category ostomy-related PIs (n = 130; 45.1%), approximately 86.9% (n = 113) were related to the ileostomy subgroup, while the proportion in the colostomy subgroup was 13.1% (n = 17). There was a statistically significant difference between the subgroups regarding the ostomy-related PIs (p = 0.025). The most frequently involved drugs were loperamide (n = 26; 20.0%) and pectin powder (n = 15; 11.5%), respectively.

### Best practice: Medication-associated ostomy-related problems (ORPs)

**Fig 2:**
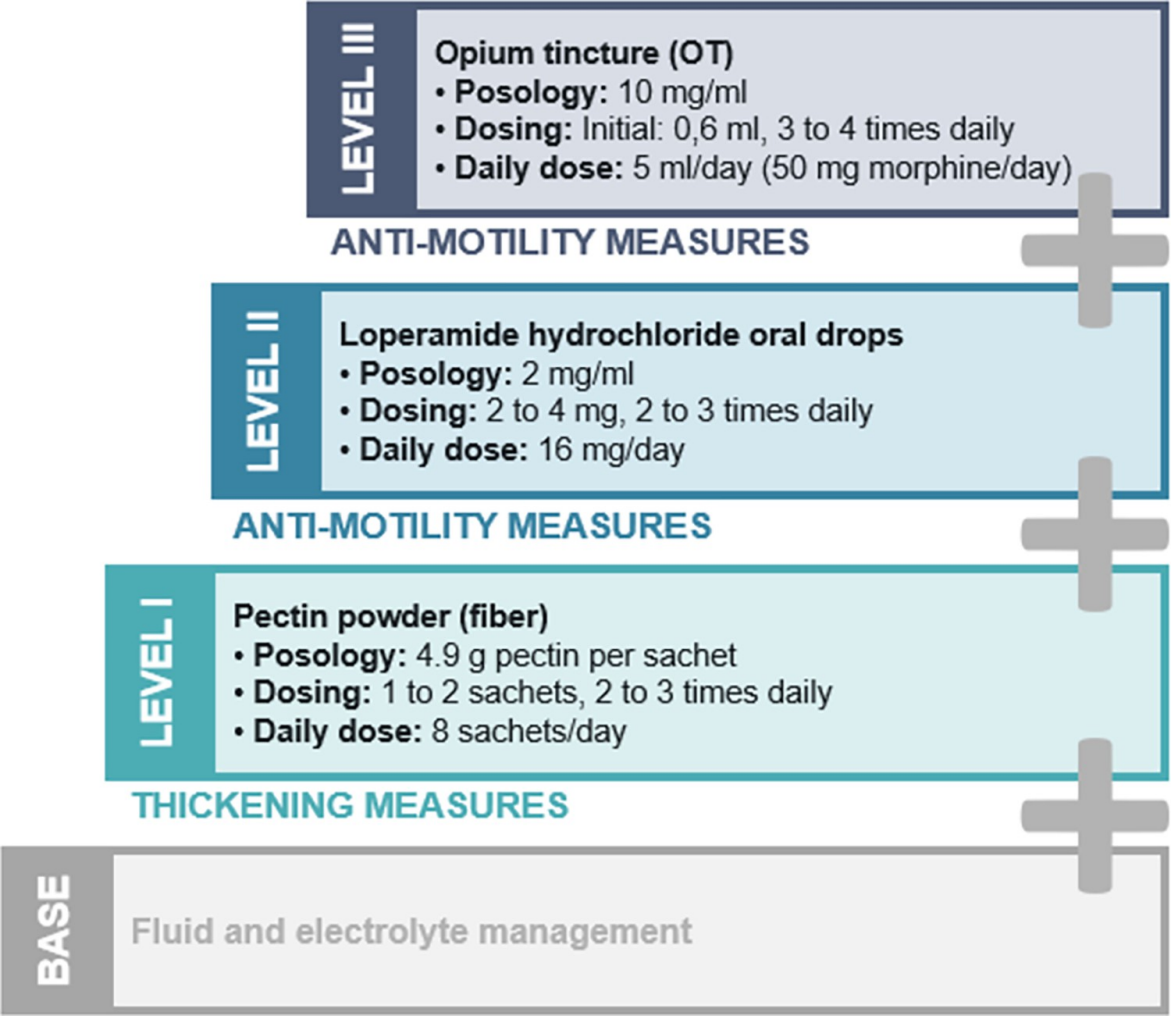
The UKE medication standard for ostomy patients with high output syndrome (HOS).

Within our study, we identified drugs that are frequently associated with ORPs. We elaborated recommendations to solve these problems with a focus on adverse drug effect profile and drug formulation. Typical clinical cases from the study highlighting potential scenarios and recommendations for optimizing drug therapy are listed in Table 4. Especially for ileostomy patients, the high output syndrome (HOS) is a clinically relevant problem, which requires early diagnosis and appropriate drug treatment. The internal hospital standard for the management of a HOS is shown in Fig 2.

**Table 4:** Typical clinical scenarios of ostomy-related problems with pharmaceutical options and recommendations.

Clinical scenario	Ostomy-related problem/ORP	Options and recommendations
***Pain management*:** **Oxycodone extended-release, ER** 58-year-old patient with a formation of a protective ileostomy treated postoperatively with a combination of immediate- and extended-release oxycodone and metamizole (dipyrone). Under current analgesia, the patient complains about pain and the oxycodone extended-release tablets appear in the ostomy bag.	Due to shortened intestine and reduced transit time, drug absorption in ileostomy patients can be reduced. The release of the drug varies depending on the drug formulation. The disintegration time for **extended-release formulations** is prolonged, so that the drug possibly is not or only partially released and absorbed.	• Before adapting the drug formulation, consider whether this patient needs an opioid therapy or whether an analgesic of WHO level 1 is sufficient. • For severe pain, it is recommended to switch to a **transdermal fentanyl patch** to achieve sufficient analgesia. Here, sufficient absorption is ensured by bypassing the gastrointestinal tract. Remember, in cachectic patients the absorption can be reduced. • Check critically in ileostomy patients with an opioid therapy if a constipation prophylaxis (e.g. macrogols) is indicated. It is often included in pain standards, but rather counterproductive in patients with high ostomy output.
***Concomitant medication*:** **Pantoprazole enteric-coated tablets** 71-year-old ileostomy patient currently suffering from high output syndrome with more than 3000 mL/day of stomal effluent. Among other drugs, the patient is treated with a proton pump inhibitor, here pantoprazole (enteric-coated tablet) to reduce gastrointestinal fluid output.	The pantoprazole enteric-coated tablet repeatedly ends up in the ostomy bag. In the context of a HOS, the transit time is often too short for **enteric-coated tablets** to dissolve.	• Do not crush pantoprazole tablets, as the enteric coating will be destroyed and efficacy will be impaired. • Especially in the hypersecretory phase after new ostomy formation, proton pump inhibitors are indicated. • Recommended aut-simile exchange to **esomeprazole tablets (Nexium® mups)**. Due to enteric coated micropellets, it is possible to suspend these tablets in water before administration. • For reasons of cost, pantoprazole tablets are often prescribed as standard.
***Nausea and vomiting*:** **Metoclopramide** 42-year-old patient complains of persistent nausea and is treated with metoclopramide. The patient’s underlying disease is ulcerative colitis and several years ago an ileostomy was placed. Currently, the ostomy carries very thin effluent and high volume.	Metoclopramide has an antiemetic effect via blockage of dopamine receptors (mainly D_2_) and serotonin receptors (5-HT_3_). At the same time, it has a **propulsive effect** via an increasing muscle motility (5-HT_4_) and a decreasing tension of the pylorus. The propulsive effect increases ostomy output.	• Postoperative nausea and vomiting (PONV) is a common problem during hospitalization. • The side effects of drugs are often unknown or disregarded until a problem occurs. • Possible alternative for postoperative nausea is **ondansetron** - ideally in form of an orally disintegrating tablet (ODT). Here, the constipating adverse drug effect can be beneficial in the case of high stomal effluent.
***Anti-motility measures*:** **Loperamide** 68 year old patient with an ileostomy and currently suffering from high output syndrome (HOS). Therapy is based on pectin powder (Aplona®) one sachet three times daily and loperamide as hard capsules with a dosage of 4 mg three times a day.	If the ostomy output is considerably increased, both thickening and motility-inhibiting measures are required. In general, better absorption is possible with **liquid formulations**, as the active drug is already present in solution. Thus the absorption process can start immediately without the need for release from the dosage form (so called "liberation").	• Loperamide is available in the form of hard capsules, ODT, drops and oral solution. The switch to a **liquid loperamide** form is preferable. • For liquid formulations, the concentration and volume must be critically examined. Especially with oral solution approved for children, high volumes and additives such as propylene glycol and sweeteners can cause laxative effects. • ODTs are also possible, but more expensive than oral solutions or hard capsules. After liberation Loperamide is absorbed via small intestine and not via the oral mucosa. • Alternatively hard capsules can be opened and stirred into water (off-label use). • Check critically concomitant medication e.g. diuretics or propulsive agents.
Summary of medication management in ostomy patients: • Consider the type of ostomy ileo- *vs*. colostomy and the duration since ostomy surgery (new *vs*. existing ostomy). • Avoid modified release drug formulations in ileostomy patients (extended release tablets/capsules, enteric-coated tablets/capsules, matrix tablets). • Prefer immediate release drug formulations (drops, sublingual tablets, effervescent tablets) or parenteral drug formulations (transdermal patches, injections/infusions). • Consider concomitant drug therapy and take into account the main and the adverse drug effects (e.g. metoclopramide *vs*. ondansetron against PONV). • Think of ostomy-related problems, ORPs (e.g. HOS, constipation, hypersecretion, nausea and vomiting).		

### High Output Syndrome (HOS)

The three-stage medication standard for ostomy patients with HOS (Fig 2) developed and implemented at the UKE is shown here as an example for a systematic approach. Apart from the recommendations for fluid and electrolyte (e.g. potassium, magnesium) management, the standard is based on thickening measures (pectin powder) and anti-motility measures (loperamide and opium tincture). Our data demonstrate that the three-step HOS ladder is well implemented, as at least one drug of the HOS standard was prescribed to 45 patients (56.0%) during hospitalization. Primarily, these were ileostomy patients, except for one colostomy patient with a short bowel syndrome. The majority of patients (n = 18) received pectin powder in combination with loperamide (level 2). The triple therapy (level 3) was indicated in 13 patients. There was no patient with opium as a monotherapy.

## Discussion

The current study, conducted in a German university hospital, evaluated an intensified medication management of ostomy patients to determine the extent of PIs and to identify DRPs in this special group of patients. Typical, recurring problems from clinical practice were outlined along with the pharmaceutical recommendations as best practice examples. We identified more PIs with a relation to the ostomy for ileostomy patients. The findings of our study suggest that both ileo- and colostomy patients benefit from a pharmaceutical medication management, but ileostomy patients in particular are more vulnerable for problems of drug absorption or HOS.

Intestinal ostomy surgery is a common surgical procedure, with more than 30,000 cases performed annually in Germany [40]. Nevertheless, we are unable to derive the actual number of ostomy patients. Over ten years ago, the number of stoma (ileo-, colo- or urostomies) patients in Germany was estimated at 160,000 [41], but there are no reliable updated numbers. Within our study, cancer was the main indication for ostomy formation, which is consistent with other studies [12, 42]. The number of prescriptions increased by a median of five drugs during hospitalization in both ileo- and colostomy group. In this context, the risk of DRPs increases with a higher number of medications [43].

To the best of our knowledge, there is no study or systematic approach, which focuses on PIs performed within a group of ostomy patients. A first exploratory analysis within our hospital demonstrated beneficial effects of an intensified medication management for ostomy patients [14]. While the literature advocates interdisciplinary collaboration in pre- and postoperative education, focusing on ostomy care, ostomy complications, diet plans or quality of life concerns, there is little information on the role of pharmacists in supporting the evaluation of drug therapy [9, 11, 17, 44]. Based on a nationwide survey in Germany, 91.0% of ostomy patients seek advice from their physician when they have problems with their medication [14]. Only a few sources mention pharmacists in connection with DRPs or switching drug formulations [11, 12, 45]. However, the Registered Nurses’ Association of Ontario (RNAO) best practice guideline, which recognizes pharmacists as key members of the interprofessional team, is an exception [18]. As a topic outside of the scope of that document pharmacists’ interventions for the prevention and management of ostomy-related complications are mentioned. The simple remark in the RNAO guideline indicates that it is a relevant problem in clinical practice.

The importance of medication management for ostomy patients is shown by the evaluation of the PIs. Compared to the median number of PIs performed as part of the routine CLMM process, there were over three additional PIs per ostomy patient case, with a higher proportion for ileostomy patients. Medication reviews were performed on at least two days during the study. In more complex patient cases with a long hospital stay, PIs were still carried out in the following weeks due to queries or problems. The high intervention rate is comparable to the study by Hilgarth et al., where clinical pharmacy services were performed in critical care units in Germany and the intervention rate was 1 PI per patient case [46]. In the PROTECTED-UK study, which also analyzed PIs in critical care units in UK, the average intervention rate was 1.2 per patient [47]. Here, the higher intervention rate is likewise explained by the more complex patient population. A recent study from Vietnam, which included a defined patient population of hypertensive outpatients and likewise provided patient education, reported 1.9 PIs per patient–without reporting how often each patient was seen [48]. The considerably higher intervention rate within our study can be explained by two aspects: Firstly, the special patient population and secondly, the intensified management over several days including level 3 medication reviews and patient interviews.

For the classification of PIs, the database DokuPIK provides differentiated reasons for PIs [49]. The proportion of PIs targeting suboptimal drug formulation is much higher in ostomy patients, particularly in ileostomy patients, compared to nationwide data on general PIs in Germany (14.9% *vs*. 4.31%) [29]. In terms of indication, literature data varies between 9.5% and 19.1% for a missing indication and in contrast for an untreated indication from 4.5% to 20.3% [24, 29, 50, 51]. Due to direct patient interviews, the rate of patient counseling is considerably high in our study. Patients had the chance to ask questions regarding their medication with no need to focus on ORPs. The category patient counseling is not included in all classification tools for PIs used in other countries [52]. The MEDAP study has demonstrated that communication-based interventions are more prevalent in outpatient settings [53]. While the proportion of interventions involving direct communication with patients was consistent with our data (8.0% *vs*. 10.0%), it is important to highlight that both inpatient and outpatient PIs were assessed in the MEDAP study. The high acceptance rate of PIs in our study results from close collaboration with physicians and a long-established clinical pharmacy service in our hospital. Working together with the WOC nurse was also essential to obtain information about problems such as increased ostomal output.

At the same time, it is important to differentiate how many PIs were exclusively related to the ostomy. A national survey of 107 ostomy patients in Germany revealed that more than half of the patients already had observed a tablet in their ostomy bag [14]. Over 70.0% of these were ileostomy patients. The occurrence of a tablet in the ostomy bag is a common problem described in the literature, especially for ileostomies, but with a lack of incidence data [9, 54, 55]. In ostomy patients, the question often arises as to what extent the absorption of drugs is impaired, due to most oral drugs being absorbed in the small intestine [56]. Zanni et al. examined the absorption profile of drugs, as well as medications that influence intestinal functioning and the risk of vitamin and mineral deficiencies [10]. Especially patients with a small bowel ostomy are more likely to have problems regarding drug absorption [57, 58]. The problem of absorption especially in ileostomy patients with regard to enteric coated or extended release tablets is also addressed by Prinz et al. in their best practice guidelines for discharge planning for patients with a new ostomy [11]. In summary, the higher proportion of ostomy-related PIs in ileostomy patients in our study confirms prior findings that ileostomy patients are more likely to suffer from problems related to drug therapy.

Furthermore, mainly ileostomy patients often experience increased ostomy output or even a high output syndrome, HOS. While clinical management of a HOS is often based on empiric data and small studies, there are no national or international guidelines. Besides fluid and electrolyte management, drug therapy is a key element in reported trials. There are only published practical approaches concerning the management of a HOS [59–64], but there is no guideline. A recent meta-analysis by Lederhuber et al. shows that there are inconsistent definitions of HOS and only limited evidence for a preferable treatment [65]. The most frequently used drugs in included studies were loperamide, somatostatin analogues and omeprazole, but there was no overall effect on stomal output to determine which intervention was most effective. The effect of opium tincture was not evaluated in the meta-analysis of Lederhuber et al., since there were no existing studies at that time. A recently published prospective, noninterventional study (CLARIFY) evaluated the therapeutic effect of opium tincture over a period of six months [66]. Main findings showed a rapid decrease of stool frequency and no observed risk for dependency after discontinuation of opium tincture. A recent randomized controlled trial by Okdahl et al. confirmed that opium tincture prolongs gastrointestinal transit time and reduces motility without signs of sedation during treatment [67]. These results are consistent with our observations from clinical practice with the internal HOS standard (Fig 2). While pectin powder in combination with loperamide was most frequently prescribed, a triple therapy with additional opium tincture was only necessary for a few patients. Although opium tincture and loperamide have the same mechanism of action (activation of intestinal μ-opioid receptors), synergistic effects have been discussed [68, 69]. The HOS standard was developed based on prescription data at our hospital [14] and is intended to function as a practical approach for other hospitals.

Several limitations of our study need to be addressed. Firstly, we included both patients with a new ostomy and patients with an existing ostomy, regardless of how long ago the ostomy was created. There was no differentiation between emergency *vs*. planned ostomy surgery. The proportion of new ostomies within our study was higher, because patients were identified in collaboration with the WOC nurse, who focuses particularly on this subgroup. In addition, more problems happen to occur in the first few weeks after ostomy formation, so here the intervention rate may be higher than in patients who have been living with an ostomy for several months or even years. However, due to the small number of patients and the very heterogeneous timespan since ostomy surgery, this trend could not be observed in our data. We categorized all ostomies as existing if they were not performed during the current hospital stay. No distinction was made about how long the patient had been living with the ostomy–whether it was one week or several years–which would be important to analyze the differences between the groups more precisely. The number of ileostomy patients was higher than the one of colostomy patients, which means that typical problems in colostomy patients may be underrepresented. Secondly, the number of medications at discharge was not recorded in detail. The patients were only asked about changes in their medication one week after discharge during interviews via telephone. After discharge, there were no medical records, only verbal information from the patients. However, the new prescriptions are particularly relevant for patients because further questions may arise in the outpatient setting.

From clinical practice, we know that problems regarding drug absorption are frequently experienced by ostomy patients. For this reason, it will be even more important to involve clinical pharmacists for medication management and patient education. Based on best practice examples obtained in this study, we aim to provide specific recommendations for frequently occurring problems in the clinical setting from a pharmaceutical perspective regarding drug therapy. Additional evaluations of data from the study regarding the relevance of pharmaceutical management and the patient’s perspective from questionnaires and interviews after discharge will be analyzed to investigate problems in the outpatient setting. The implementation of an intensified medication management to a larger number of hospitals and the measurement of patient-reported outcome are key elements for future trials.

## Conclusions

Many patients are facing problems due to ostomy formation in the context of drug therapy. However, there are options and solutions to address these problems. As specialists for drug therapy, clinical pharmacists can help to analyze and optimize the medication. Each patient, each ostomy as well as each drug therapy must be considered individually, but standards can be implemented for both the PIs and the therapeutic approaches (e.g. high output syndrome, HOS). Raising awareness amongst healthcare professionals and patients should be the first step to an overall improvement in patient safety. Subsequently, the collaboration between physicians, WOC nurses and clinical pharmacists should be enhanced. Especially, ileostomy patients benefit from an intensified medication management by clinical pharmacists.

## Supporting information

S1 Table List of drugs and reasons for classification into ostomy-related PIs. (PDF)
