# Supplementary material for: Enhancing drug therapy in ostomy patients: Best practice recommendations for medication management
Source: PLoS One. 2024 Jun 6;19(6):e0305047. doi: 10.1371/journal.pone.0305047 (PMC11156294; doi:10.1371/journal.pone.0305047)
Supplement: S1 Table — (PDF) [file pone.0305047.s001.pdf]

S1 Table: List of drugs for classification into ostomy-related PIs		
**Drugs**	**ATC classification (1st level)**	**ATC (5th level)**
omeprazole	**A: Alimentary tract and metabolism**	A02BC01
pantoprazole		A02BC02
esomeprazole		A02BC05
silicones		A03AX13
butylscopolamine		A03BB01
metoclopramide		A03FA01
domperidone		A03FA03
ondansetron		A04AA01
granisetron		A04AA02
dimenhydrinate		A04AB02
ursodeoxycholic acid		A05AA02
sodium picosulfate		A06AB08
ispaghula (psylla seeds)		A06AC01
lactulose		A06AD11
macrogol, combinations		A06AD65
prucalopride		A06AX05
opium		A07DA02
loperamide		A07DA03
pectin		A07XP05
multienzymes (lipase, protease etc.)		A09AA02
colecalciferol		A11CC05
potassium chloride		A12BA01
potassium (different salts in combination)		A12BA30
ferrous glycine sulfate	**B: Blood and blood forming organs**	B03AA01
cyanocobalamin		B03BA01
folic acid		B03BB01
potassium chloride		B05XA01
octreotide*	**H: Systemic hormonal preparations, excluding sex hormones and insulins**	H01CB02
erythromycin*	**J: Antiinfective for systemic use**	J01FA01
oxycodone	**N: Nervous system**	N02AA05
metamizole sodium		N02BB02
neostigmine*		N07AA01
codeine*	**R: Respiratory system**	R05DA04

Note: The drugs were identified in a previous project [33] and are primarily determined by the clinic medication list.

* The labeled drugs are being used off-label for indications outside the ATC classification.

Abbreviation: ATC = Anatomical Therapeutic Chemical (ATC) classification

S1 Table: List of reasons for PIs according to DokuPIK (classified in regular and ostomy-related PIs)	
**Category: Administration**	
Request/Query concerning administration/compatibility	regular
Administration (route)	regular
Administration (duration)	regular
Incompatibility or incorrect preparation or reconstitution	regular
**Category: Adverse drug reaction**	regular
**Category: Contraindication**	regular
**Category: Dose**	
Failure to adjust dose for organ dysfunction	regular
(Inappropriate) dose	ostomy-related (S1A drugs)
(Inappropriate) administration interval	ostomy-related (S1A drugs)
TDM not performed or not considered	regular
**Category: Drug**	
(Clear) indication not (or no longer) given	ostomy-related (S1A drugs)
(Clear) indication, but no drug prescribed	ostomy-related (S1A drugs)
Drug allergy or medical history not considered	regular
Double prescription	regular
Dispensing error on the ward	regular
Generic/Therapeutic substitution	regular
Transcription error	regular
Inappropriate or not most suitable drug formulation in terms of indication	ostomy-related (for all drugs)
Inappropriately or not most suitable drug in terms of costs	
Inappropriate or not most suitable drug in terms of indication	ostomy-related (S1A drugs)
Prescription/Documentation incomplete/incorrect	regular
**Category: Interaction**	regular
**Category: Other**	
Advisory service drug choice	ostomy-related (S1A drugs)
Advisory service drug dose	ostomy-related (S1A drugs)
Procurement/Costs	regular
Failure to discontinue relevant drugs pre-/perioperatively	regular
Patient counselling or education	ostomy-related (for ostomy issues)

Note: The term "S1A drugs" refers to the selection of drugs listed in the S1A Table.
Abbreviation: TDM = Therapeutic Drug Monitoring
